# Ethyl Acetate Extract of *Artemisia anomala* S. Moore Displays Potent Anti-Inflammatory Effect

**DOI:** 10.1155/2014/681352

**Published:** 2014-03-12

**Authors:** Xi Tan, Yuan-Lai Wang, Xiao-Lu Yang, Dan-Dan Zhang

**Affiliations:** Shanghai University of Traditional Chinese Medicine, Pudong, Shanghai 201203, China

## Abstract

*Artemisia anomala* S. Moore has been widely used in China to treat inflammatory diseases for hundreds of years. However, mechanisms associated with its anti-inflammatory effect are not clear. In this study, we prepared ethyl acetate, petroleum ether, *n*-BuOH, and aqueous extracts from ethanol extract of *Artemisia anomala* S. Moore. Comparing anti-inflammatory effects of these extracts, we found that ethyl acetate extract of this herb (EAFA) exhibited the strongest inhibitory effect on nitric oxide (NO) production in LPS/IFN**γ**-stimulated RAW264.7 cells. EAFA suppressed the production of NO in a time- and dose-dependent manner without eliciting cytotoxicity to RAW264.7 cells. To understand the molecular mechanism underlying EAFA's anti-inflammatory effect, we showed that EAFA increased total cellular anti-oxidant capacity while reducing the amount of inducible nitric oxide synthase (iNOS) in stimulated RAW264.7 cells. EAFA also suppressed the expression of IL-1**β** and IL-6, whereas it elevates the level of heme oxygenase-1. These EAFA-induced events were apparently associated with NF-**κ**B and MAPK signaling pathways because the DNA binding activity of p50/p65 was impaired and the activities of both ERK and JNK were decreased in EFEA-treated cells comparing to untreated cells. Our findings suggest that EAFA exerts its anti-inflammatory effect by inhibiting the expression of iNOS.

## 1. Introduction

*Artemisia anomala *S. Moore (Nan-Liu-Ji-Nu) is a perennial herbaceous plant categorized to* Artemisia* genus Compositae family. Many species of* Artemisia* have been used as medicinal materials. In fact,* Artemisia anomala* S. Moore has been used for centuries to treat fever, empyrosis, inflammation, and dissipated liver function caused by hepatitis in China. For example,* Artemisia* oil can potently inhibit the growth of bacteria, yeasts, dermatophytes, and* Aspergillus niger* and has thus been extensively used as an anti-inflammatory agent [1]. The most well-known medicine from* Artemisia* genus is probably artemisinin and its derivatives that have the rapidest action against malaria among all antimalaria drugs. The regiment containing at least one artemisinin derivative (artemisinin-combination therapies) is the standard protocol to treat* P. falciparum* malaria worldwide [2]. The therapeutic effect of* Artemisia anomala *S. Moore is likely to be linked to its ability to counteract against inflammation, oxidation [3], and viral infection [4]. Recent studies show that dimeric guaianolides and sesquiterpenoids extracted from the aerial part of* Artemisia anomala *can suppress cyclooxygenase 2- (COX2-) associated effects [5]. Commonly used prostaglandin-like fatty acid derivatives anomalone A-D were actually isolated from* Artemisia anomala *[6]. Although sufficient evidences have demonstrated* Artemisia anomala *S. Moore as an effective anti-inflammatory agent, systematically evaluating its anti-inflammatory effects with inflammatory parameters has yet been performed.

Acute inflammatory response represents an initial protective mechanism in the body. In contrast, excessive and chronic inflammation results in severe damages of cells and tissues. Emerging evidences support the hypothesis that chronic inflammation plays a critical role in various pathological conditions, including hypertension, atherosclerosis, stroke, metabolic diseases, cancer, autoimmune disorders, and neurodegenerative diseases [7–10]. Nitric oxide (NO) is a free radical that is synthesized from L-arginine by nitric oxide synthase (NOS). There are three types of NOS: two constitutive NOS, eNOS and nNOS, and one inducible NOS (iNOS). Constitutive NOSs generate nanomolar concentration of NO and are known to mediate various physiological functions. Contrarily, iNOS produces NO at the level of micromolar that often results in pathological consequences such as chronic inflammation. Inflammatory stimuli can induce iNOS expression through distinct signaling pathways. Proinflammatory cytokines released from inflammation-stimulated cells, for example, macrophages, can further upregulate iNOS expression and augment inflammatory responses [11, 12]. The expression of proinflammatory cytokines, including interleukin-1*β* (IL-1*β*), interleukin-6 (IL-6), is often regulated through the NF-*κ*B and MAPK signaling pathways [13, 14]. Endogenous anti-inflammatory response is also involved in inducible heme oxygenase-1 (HO-1). Because of the ability of HO-1 to attenuate iNOS expression [15, 16], HO-1 is thought to play a protective role during inflammation [17, 18].

The objective of this study is to determine the most effective fraction of* Artemisia anomala* S. Moore that can inhibit iNOS-induced NO production. To identify such fraction, we prepared ethyl acetate, petroleum ether,* n*-BuOH, and aqueous extracts from ethanol extract of* Artemisia anomala* S. Moore. With the aid of the well-established murine macrophage RAW264.7 cell inflammation model, we found that ethyl acetate extraction of* Artemisia anomala* S. Moore (EAFA) exhibited the strongest inhibitory effect on LPS/IFN*γ*-induced NO production and proinflammatory cytokine expression. Since NF-*κ*B and MAPK activities were significantly reduced in EAFA-treated cells, we suggest that EAFA exerts its inhibitory action by interfering with both NF-*κ*B and MAPK signaling pathways.

## 2. Materials and Methods

### 2.1. Reagents

Murine recombinant IFN*γ*, NF-*κ*B p50/p65 EZ-TFA transcription factor assay system, and mouse IL-6 ELISA kit were purchased from Millipore (MA, USA); lipopolysaccharide (LPS,* Escherichia coli* O111:B4), dimethyl sulfanilamine,* N*-(1-naphthyl)-ethylenediamine dihydrochloride, 3-(4,5-dimethylthiazol-2-yl)-2,5-diphenyltetrazoleum (MTT), L-N^6^-(1-Iminoethyl)lysine hydrochloride (L-NIL), and Trolox were obtained from Sigma (St. Louis, MO). TRIzol Reagent was obtained from Invitrogen (Carlsbad, CA). Mouse IL-1beta instant ELISA was obtained from eBioscience (San Diego, CA). Takara SYBR kit and OligodT were obtained from Shanghai Invitrogen (Shanghai, China). Nuclear Extraction Kit was obtained from Biyuntian (Shanghai, China). Antibodies used in this study include murine iNOS monoclonal antibody from BD Transduction Laboratories (Lexington, KY); murine *β*-actin and HO-1 antibodies from Santa Cruz Biotechnology (Santa Cruz, CA); phosphor-p38, p38, phosphor-JNK, JNK, and phosphor-ERK1/2 and ERK1/2 antibodies from Cell Signaling Technology (Danvers, MA).

### 2.2. Herb Extraction and Fractionation

*Artemisia anomala* S. Moore was purchased from Yang-He Tang Co. (Zhangjiang High-Tech Park, Shanghai, China) and confirmed by Shanghai Institute for Food and Drug Control (SIFDC). The dried plants were first extracted with 70% ethanol at 80°C for three times (200 g raw material/1 L/60 min each time) and the obtained ethanol extract was then suspended in water followed by the constitutive partition with petroleum ether, ethyl acetate,* n*-butanol, and water. After evaporation of these partitioned solutions, five extract fractions were generated: ethanol (yield 6.73%), petroleum ether (0.17%), ethyl acetate (0.25%),* n*-butanol (0.33%), and aqueous fractions (1.64%). Each fraction was dissolved in DMSO and stored at −20°C until use.

### 2.3. Cell Culture

RAW264.7 cells were originally obtained from the American Tissue Culture Collection. Cells were maintained in RPMI 1640 medium supplemented with 10% FBS at 37°C in a humidified 5% CO_2_ atmosphere.

### 2.4. Measurement of NO Production

RAW264.7 cells were plated in a 96-well plate (5 × 10^3^ cells/well) for overnight and then serum-starved for 10 h followed by the addition of 10 U/mL IFN*γ* and 100 ng/mL LPS for 24 h in the presence or absence of different* Artemisia anomala* S. Moore fractions with finally concentration at 10, 100, 200 *μ*g/mL and used L-NIL (50 *μ*M) as positive drug control for primary screening. After obtaining the strongest fraction, the posttreat, pretreat, and simultaneous-treat of this fraction and stimulation would underprocess for secondary screening. To analyze NO production, 100 *μ*L of supernatant was incubated with equal volume of Griess solution at room temperature for 10 min and absorbance was then read at 540 nm. Since NO content was reflected by the amount of nitrite, a calibration curve was generated using sodium nitrite. The amount of nitrite in the supernatants was calculated based on the calibration curve. The percentage inhibition of NO production is evaluated using the formula {1−[(nitrite amount of fraction – treated)/(nitrite amount of vehicle)]} × 100.

### 2.5. Assay for Cell Viability

Cell viability was assessed by MTT assay. Briefly, after using the 100 *μ*L supernatants to do Griess reaction, the rest cells were incubated with 10 *μ*L MTT (5 mg/mL in phosphate-buffered saline, pH = 7.4) for 4 h at 37°C followed by adding 50 *μ*L 0.01 mol/L HCl buffer containing 10% SDS and 10% Isopropanol. Absorbance was measured at 540 and 630 nm in a microplate reader. The absorbance of control (untreated) cells was considered as 100% of viability.

### 2.6. Measurement of Total Antioxidant Capacity

Total antioxidant activity was measured by modified FRAP assay as previously described [19]. Briefly, RAW264.7 cells (2 × 10^6^ cells/30 mm dish) were pretreated with varying concentration of EAFA (50, 100, 200 *μ*g/mL) for 1 h followed by costimulation of 100 ng/mL LPS and 10 U/mL IFN-*γ* for 6 h. Cells were harvested, whereas the supernatants were collected. FRAP reagent was prepared by mixing 300 mmol/L acetate buffer (pH 3.6), 10 mmol/L 2,4,6-tripyridyl-s-triazine (TPTZ) in 40 mmol/L HCl solution and 20 mmol/L FeCl_3_ in a 10 : 1 : 1 ratio and 245 *μ*L of freshly prepared FRAP solution was added to each well of a 96-well plate that contained 5 *μ*L of supernatant. After 10 min incubation at room temperature, absorbance was measured at 593 nm with the aid of a microplate reader. A standard curve was prepared with various concentrations of Trolox (0.03125 to 2 mmol/L). The potency of total antioxidant capacity for each sample was determined by comparing the antioxidant capacity of 1 mM Trolox.

### 2.7. Detection of IL-1*β* and IL-6 in Supernatant

Inhibitory effects of EAFA on the cytokine IL-6 and IL-1*β* production from LPS plus IFN-*γ* treated RAW264.7 cells were detected by sandwich ELISA. The procedure was carried out under the instructions from respective kit. After preincubation of 1 h with different dosage of EAFA and stimulation with LPS plus IFN-*γ* on RAW264.7 cells for 24 h, supernatants were harvested and assayed for IL-1*β* and IL-6. Results of three independent experiments were used for statistical analysis.

### 2.8. RNA Isolation and Quantitative RT-PCR

Total RNA was isolated using TRIzol Reagent according to manufacturer's instruction. Quantitative RT-PCR (qRT-PCR) was performed with Takara SYBR kit using the primers sets in Table 1 as previously described [20]. The 2^−ΔΔCT^ method was utilized to analyze the fold increase.

### 2.9. Nuclear Extract Preparation and NF-*κ*B DNA Binding Assay

Nuclear extracts were prepared using Nuclear Extraction Kit according to the manufacturer's instruction. DNA binding activity of NF-*κ*B in nuclear extracts was assessed using NF-*κ*B p50/p65 EZ-TFA transcription factor assay kit which detects the amount of NF-*κ*B in the nucleus.

### 2.10. Statistical Analysis

Student's test was used to analyze the difference between treated and untreated groups. Comparisons between multiple groups were performed with one-way ANOVA test. *P* < 0.05 was considered statistically significant.

## 3. Results

### 3.1. EAFA Displays the Strongest Inhibitory Effect on LPS/IFN*γ*-Induced NO Production

**Figure 1:**
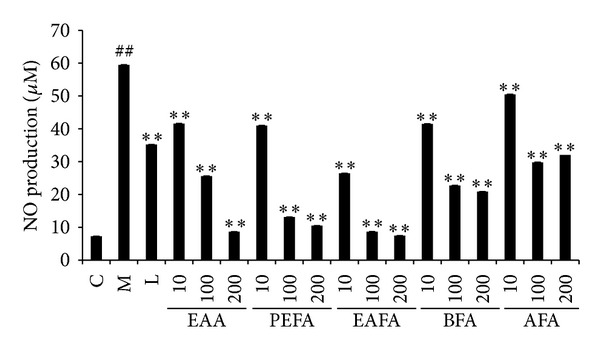
Effect of four solvent extracts of ethanol extract of* Artemisia anomala* S. Moore on NO production in LPS/IFN*γ*-stimulated RAW264.7 cells. C: control (nontreatment) group; M: LPS/IFN*γ*-stimulated model group; L: L-NIL (50 *μ*M); EAA: ethanol extracted fraction (10, 100, 200 *μ*g/mL); PEFA: petroleum ether extracted fraction (10, 100, 200 *μ*g/mL); EAFA: ethyl acetate extracted fraction (10, 100, 200 *μ*g/mL). BFA:* n*-butanol extracted fraction (10, 100, 200 *μ*g/mL); AFA: queous extracted fraction (10, 100, 200 *μ*g/mL). Data are mean ± SEM *n* = 6 per group. **P < 0.01 (*versus* model group). ^##^ *P* < 0.01 (*versus* control group).

The ethanol extract of* Artemisia anomala* S. Moore has been shown to exhibit inhibition of NO production in our previous screening [21]. We extracted ethanol extract of* Artemisia anomala* S. Moore further with petroleum ether, ethyl acetate,* n*-butanol, and water, and each of these obtained extracts was tested for its ability to inhibit NO production in RAW264.7 cells costimulated with LPS and IFN*γ*. Griess reaction assay showed that IC_50_ of original ethanol extract (EEA) was 31.07 *μ*g/mL (Figure 1). IC_50_ of petroleum ether fraction (PEFA),* n*-butanol fraction (BFA), and aqueous fraction (AFA) from the EEA was 21.73, 39.10, and 49.25 *μ*g/mL, respectively (Figure 1), which were similar to that of the original EEA. In contrast, IC_50_ of ethyl acetate fraction (EAFA) was 15.85 *μ*g/mL (Figure 1), representing twice stronger inhibitory effect over the original EEA.

**Figure 2:**
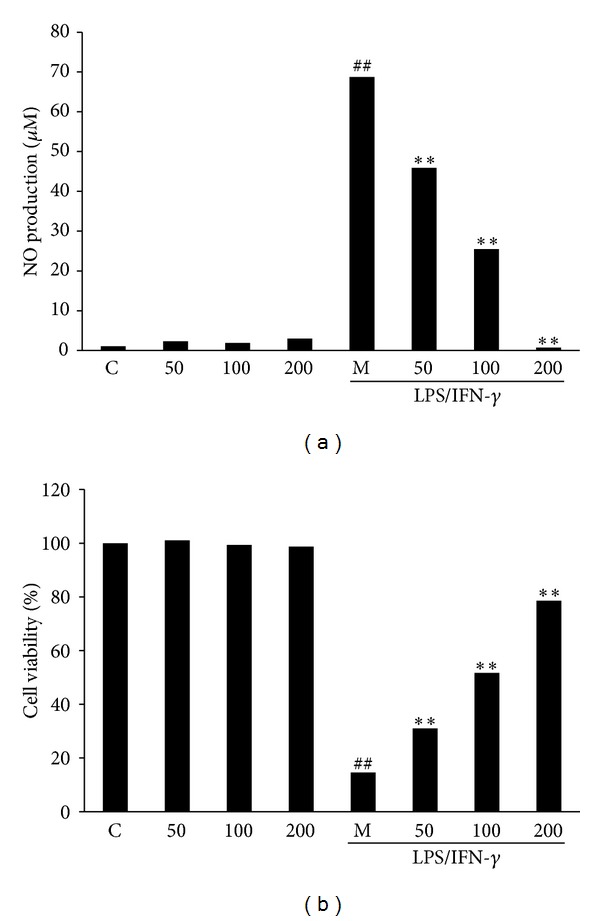
Effect of EAFA on NO production (a) and cell viability (b) in LPS/IFN*γ*-stimulated and unstimulated RAW264.7 cells. RAW264.7 cells were treated with EAFA (50–200 *μ*g/mL) for 24 h with or without LPS/IFN*γ* stimulation. Nitrite concentrations in the culture medium were determined by the Griess reaction. Changes in survival are represented as percentages of the control group. Bars represent the mean ± SEM. Three independent experiments were performed. ^##^ *P* < 0.01; ^#^ *P* < 0.05* versus* control group; **P < 0.01; *P < 0.05* versus* model group.

In a parallel experiment, we investigated the effect of EAFA on NO production in unstimulated RAW264.7 cells. Contrary to its ability to dose-dependently inhibit NO production in LPS/IFN*γ*-stimulated cells (Figure 2(a)), EAFA displayed little effect on NO production in unstimulated cells (Figure 2(a)). MTT assays further showed that EAFA promoted viability of LPS/IFN*γ*-stimulated RAW246.7 cells in dose-dependent manner while it exhibited effect on the viability of unstimulated cells (Figure 2(b)). These results suggest that EAFA selectively inhibits NO production. Since EAFA promotes cell viability in LPS/IFN*γ*-stimulated RAW264.7 cells (Figure 2(b)), these results also indicate that the inhibitory effect of EAFA on NO production in LPS/IFN*γ*-stimulated cells is not caused by LPS/IFN*γ*-induced cellular toxicity.

### 3.2. Both Pre- and Posttreatments of EAFA Inhibit LPS/IFN*γ*-Induced NO Production and Cellular Toxicity in RAW264.7 Cells

**Figure 3:**
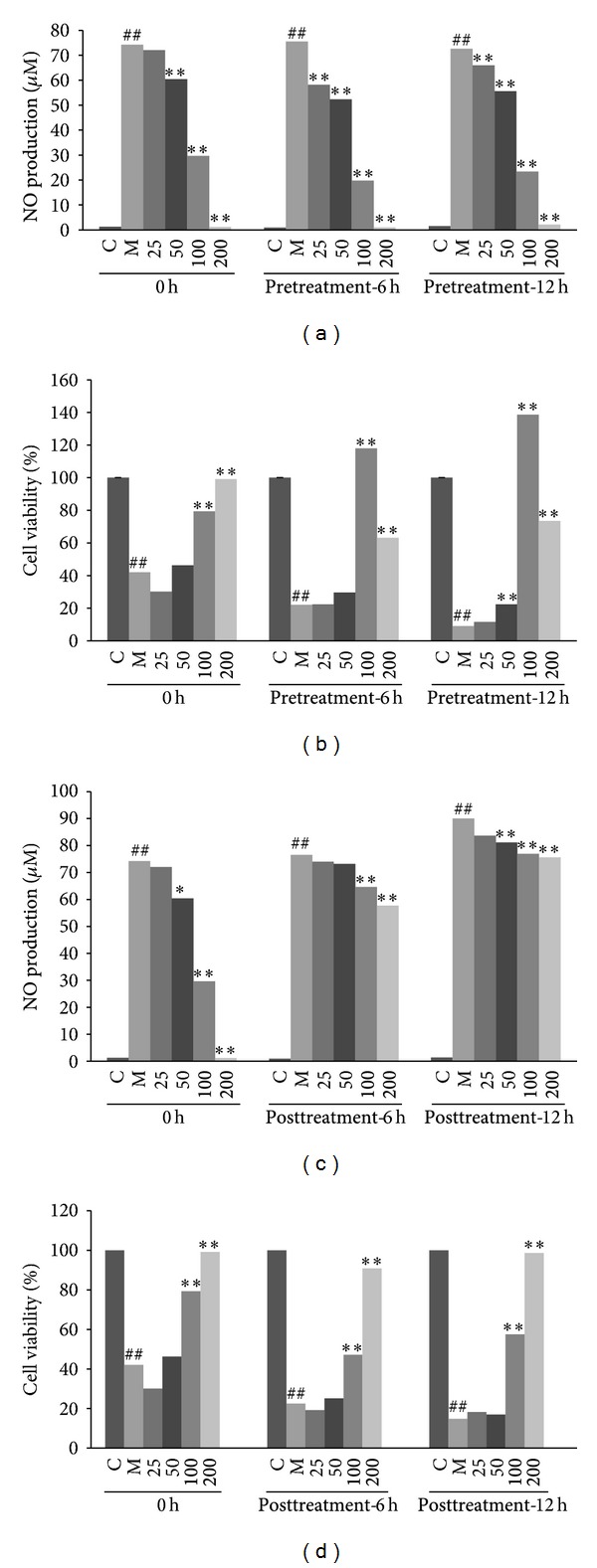
Effect of pretreatment and posttreatment of EAFA on NO production and cell viability. Cells were plated at a density of 1 × 10^5^ cells/well in a 96-well plate and allowed to attach for 2 h. EAFA was added prior to (pretreatment-12 h, pretreatment-6 h), simultaneously with (0 h) (a, b) or after the treatment of the cells with IFN*γ* (10 U/mL) plus LPS (100 ng/mL) (posttreatment-6 h, posttreatment-12 h) (c, d). Nitric concentrations in the culture medium and cell viability were determined by the Griess reaction and MTT assay. The values (means ± SEM) were obtained from three independent experiments. ^##^ *P* < 0.01; ^#^ *P* < 0.05* versus* control group; **P < 0.01; *P < 0.05 * versus* model group.

To further characterize the pharmacological action by EAFA, we pretreated RAW264.7 cells with EAFA for 6 and 12 hrs followed by LPS/IFN*γ* stimulation. Griess reaction assays showed that EAFA pretreatment resulted in significantly better inhibitory effect on NO production in LPS/IFN*γ*-stimulated RAW264.7 cells than adding EAFA at the time of LPS/IFN*γ* stimulation (Figure 3(a)). Similarly, EAFA pretreatment promoted cell viability in a greater degree than adding EAFA simultaneously with the stimulants (Figure 3(b)). In subsequent study, RAW264.7 cells were first stimulated with LPS/IFN*γ* for 6 or 12 h and then treated with EAFA. Although less inhibitory effect on NO production and cell viability was detected with EAFA posttreatment compared with EAFA pretreatment, we still observed 16.22% of reduction in NO production and 98.58% of increase in cell viability in RAW264.7 cells treated with EAFA at dosage of 200 *μ*g/mL (Figures 3(c) and 3(d)). Taken together, these results suggest than EAFA can potentially be used both as a preventive and therapeutic agent against chronic inflammation.

### 3.3. EAFA Pretreatment Prevents LPS/IFN*γ*-Suppressed Antioxidant Capacity and Inhibits iNOS Expression

**Figure 4:**
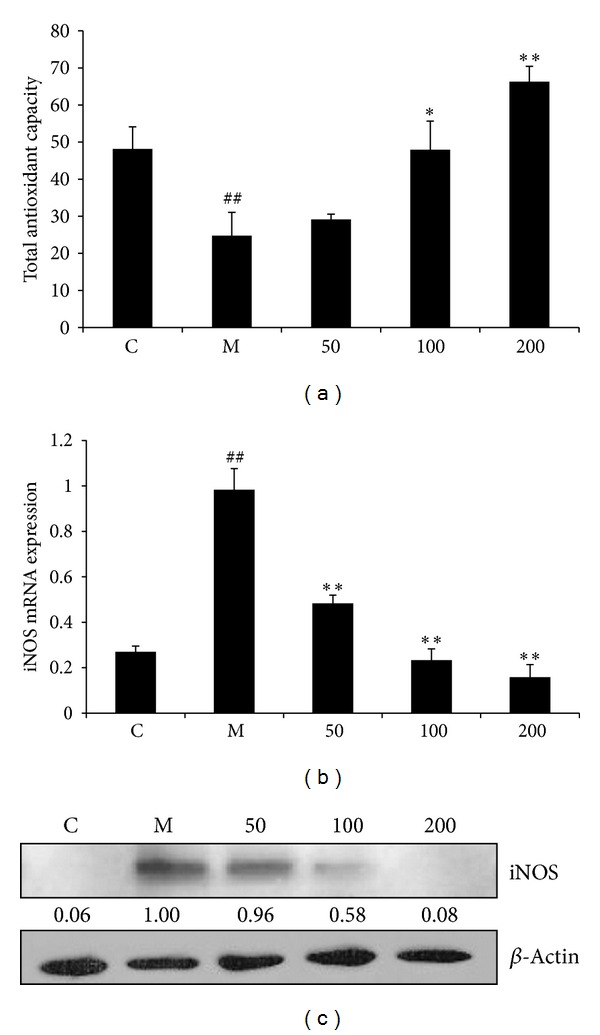
Effect of EAFA on total oxidant activity and iNOS expression in LPS/IFN*γ*-stimulated RAW264.7 cells. (a) Cells were plated at a density of 1 × 10^6^ cells/well in 30 mm dishes and allowed to attach overnight. EAFA was added 1 h prior to the treatment of IFN*γ* (10 U/mL) plus LPS (100 ng/mL) for 6 h. Whole cell lysates were analyzed by FRAP assay and standardized by protein concentration. The data shown are representative of three independent experiments. ^##^ *P* < 0.01; ^#^ *P* < 0.05* versus* control group; **P < 0.01; *P < 0.05* versus* model group. (b) RAW264.7 cells (1 × 10^6^ cells/dish) were pretreated with varying concentrations of EAFA for 1 h followed by LPS (100 ng/mL) and IFN-*γ* (10 U/mL) treatment for 4 h. Total RNA was isolated and subjected to qRT-PCR. *β*-actin mRNA was used as an internal control for standardization. (c) RAW264.7 cells were plated at a density of 1 × 10^6^ cells in 30 mm dish for overnight. EAFA was added 1 h prior to the treatment of IFN*γ* (10 U/mL) plus LPS (100 ng/mL) for 6 h. Whole cell lysates were prepared and subjected to western blotting. The data shown are representative of three independent experiments. ^##^ *P* < 0.01, ^#^ *P* < 0.05* versus* control group; **P < 0.01, *P < 0.05* versus* model group.

NO at high concentration is often considered as oxidant stress [22]. Since EAFA can effectively reduce LPS/IFN*γ*-induced NO production, we hypothesized that EAFA might also possess potent antioxidant activity. To test this hypothesis, RAW264.7 cells were pretreated with varying concentrations of EAFA for 1 h followed by LPS/IFN*γ* stimulation for 6 h. Total ferric reducing-antioxidant power (FRAP) assay showed that LPS/IFN*γ* stimulation greatly reduced antioxidant capacity (TAC) in RAW264.7 cells (Figure 4(a)). However, EAFA pretreatment reversed LPS/IFN*γ*-caused reduction in TAC (Figure 4(a)).

The fact that iNOS is responsible for LPS/IFN*γ*-induced NO production indicates that EAFA might block NO production by decreasing the amount of iNOS. Because iNOS is mainly regulated at transcription level [23], we tested this possibility by determining the effect of EAFA on iNOS mRNA in LPS/IFN*γ*-stimulated RAW264.7 cells with the aid of quantitative RT-PCR (qRT-PCR). LPS/IFN*γ* stimulation elevated the level of iNOS; however, pretreatment of EAFA at 100 and 200 *μ*g/mL led to 41.78% (*P* < 0.01) and 85.29% (*P* < 0.01) reduction in LPS/IFN*γ*-induced iNOS expression (Figure 4(b)). Western blot analysis also showed that EAFA pretreatment diminished LPS/IFN*γ*-induced iNOS protein expression in RAW264.7 cells (Figure 4(c)). Taken together, these results support the notion that EAFA is a potent preventive agent against inflammation.

### 3.4. EAFA Blocks Inflammatory Cytokines Production and Increases HO-1 Expression in RAW246.7 Cells

**Figure 5:**
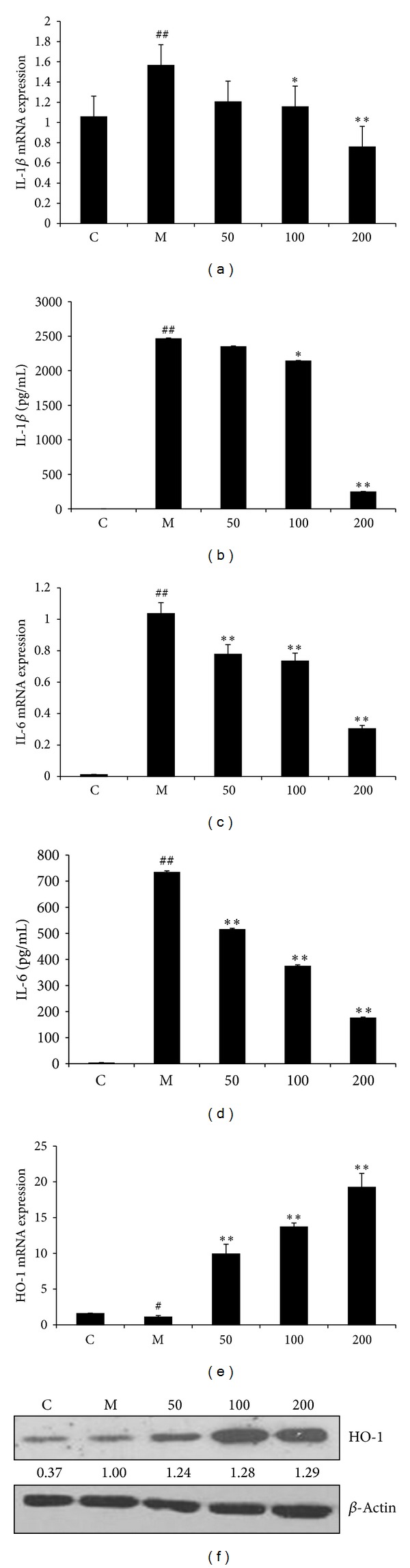
Effect of EAFA on proinflammatory cytokine and HO-1 expression in LPS/IFN*γ*-stimulated RAW264.7 cells. (a, b) RAW264.7 cells (1 × 10^6^ cells/dish) were pretreated with varying doses of EAFA for 1 h and then stimulated with IFN*γ* (10 U/mL) plus LPS (100 ng/mL) for 6 h. Total RNA was isolated and subjected to qRT-PCR to determine the level of IL-1*β* (a) and IL-6 mRNA (b). (c, d) RAW264.7 cells were treated with IFN*γ* (10 U/mL) plus LPS (100 ng/mL) in the presence of varying concentrations of EAFA for 24 h. Conditioned media were collected and subjected to ELISA to determine the amount of IL-1*β* (c) and IL-6 (d). The values (means ± SEM) were obtained from three independent experiments. ^##^ *P* < 0.01, ^#^ *P* < 0.05* versus* control group; **P < 0.01, *P < 0.05* versus* model group. (e) RAW264.7 cells (1 × 10^6^ cells/dish) were pretreated with varying concentrations of EAFA (50, 100, 200 *μ*g/mL) for 1 h followed by LPS (100 ng/mL) plus IFN*γ* (10 U/mL) treatment for 18 h. Total RNA was isolated and subjected to qRT-PCR to measure HO-1 mRNA level. *β*-actin was used as an internal control for standardization. (f) RAW264.7 cells plated at a density of 1 × 10^6^ cells/well in 30 mm dish for overnight were pretreated with EAFA for 1 h followed by 18 h-stimulation of IFN*γ* (10 U/mL) plus LPS (100 ng/mL). Whole cell lysates were prepared and subjected to western blotting to determine HO-1 protein levels. Data are the representative of three independent experiments. ^##^ *P* < 0.01, ^#^ *P* < 0.05* versus* control group; *P < 0.01, **P < 0.05* versus* model group.

LPS/IFN*γ* costimulation has been reported to induce the expression of a plethora of proinflammatory cytokines in macrophages [24]; we thus investigated the effect of EAFA on IL-1*β* and IL-6 expressions in LPS/IFN*γ*-stimulated RAW246.7 cells. qRT-PCR showed that LPS/IFN*γ* stimulation led to over 1.5-fold increase in IL-1*β* and 104-fold increase in IL-6 expression. Pretreatment of EAFA dose-dependently abrogated LPS/IFN*γ*-induced IL-1*β* and IL-6 expression (Figures 5(a) and 5(c)). ELISA with the conditioned media also showed that EAFA pretreatment diminished LPS/IFN*γ*-induced IL-1*β* and IL-6 secretion by RAW246.7 cells (Figures 5(b) and 5(d)). The nature of HO-1 as a stress-inducible protein with anti-inflammatory feature [17, 18] also prompted us to determine how EAFA affected HO-1 expression in LPS/IFN*γ*-stimulated RAW246.7 cells. Western blot analysis showed that EAFA pretreatment upregulated HO-1 abundance while qRT-PCR revealed that EAFA increased the level of HO-1 mRNA in a dose-dependent manner (Figures 5(e) and 5(f)). These results indicate that the ability of EAFA to prevent inflammatory responses was two-folded: one is to abolish inflammatory cytokine expression and the other is to increase HO-1 expression in macrophages.

### 3.5. NF-*κ*B and MAPK Signaling Pathways Are the Target of EAFA-Mediated Inhibition

**Figure 6:**
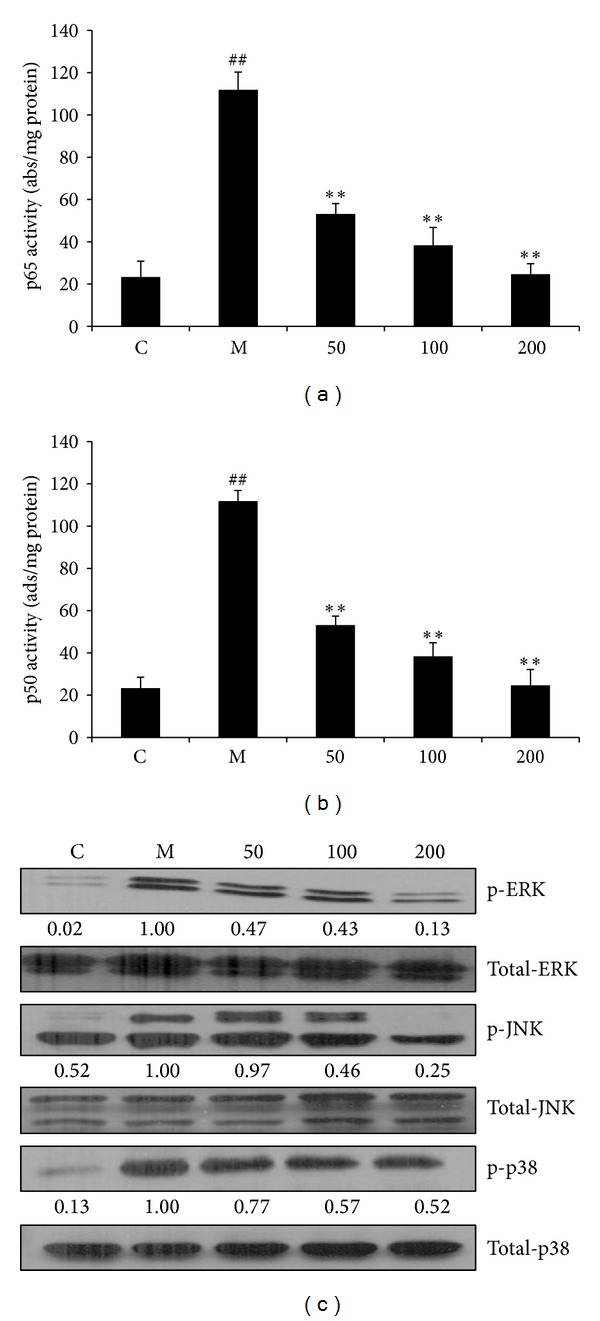
Effect of EAFA on NF-*κ*B and MAPK activities in LPS/IFN*γ*-stimulated RAW264.7 cells. (a, b) DNA binding activity of p50 and p65 proteins in nuclear extracts was assessed using NF-*κ*Bp50/p65 EZ-TFA transcription factor assay. Absorbance was measured at 450 nm in a microplate spectrophotometer. Results were normalized to absorbance/mg protein. The data shown are representative of three independent experiments. ^##^ *P* < 0.01, ^#^ *P* < 0.05 * versus* control group; **P < 0.01, *P < 0.05 * versus* model group. (c) RAW264.7 cells were plated at a density of 1 × 10^6^ cells/well in 30 mm dish for overnight. EAFA was added to cells for 1 h followed by 30 min stimulation of IFN*γ* (10 U/mL) plus LPS (100 ng/mL). Whole cell lysates were prepared and subjected to western blotting to detect phosphor-ERK, phosphor-JNK, and phosphor-p38 with the respective antibodies. Data shown are the representative of three independent experiments. ^##^ *P* < 0.01, ^#^ *P* < 0.05 * versus* control group; **P < 0.01, *P < 0.05 * versus* model group.

NF-*κ*B activity is known to be critical for the expression of iNOS [25–27]. To investigate how EAFA affected LPS/IFN*γ*-induced NF-*κ*B activity, we analyzed the extent of p50 and p65 binding to NF-*κ*B consensus sequence-containing oligonucleotides in nuclear extracts. LPS/IFN*γ* stimulation resulted in more than 5-fold increase in the amount of p50 and p65 bound to the NF-*κ*B consensus sequence-containing oligonucleotides compared with unstimulated RAW246.7 cells (Figures 6(a) and 6(b)). However, EAFA inhibited LPS/IFN*γ*-induced NF-*κ*B activation and, at 200 *μ*g/mL, completely abolished this activation (Figures 6(a) and 6(b)). In addition to NF-*κ*B, members of MAPK families have also been implicated to play an essential role in the inflammatory reaction. To determine the effect of EAFA on LPS/IFN*γ*-induced MAPK activation, western blots were performed to analyze the levels of phosphor-Erk, JNK, and p38 in RAW246.7 cells. LPS/IFN*γ* stimulation (30 min) evocated significant increases in the levels of phosphorylated Erk, JNK, and p38 in RAW246.7 cells. However, pretreatment of EAFA markedly inhibited the extent of Erk and JNK phosphorylation (Figure 6(c)). These results suggest that EAFA blocks inflammatory responses by the combination of blocking NF-*κ*B, Erk, and JNK activation.

## 4. Discussion

Inhibition of iNOS has been shown to soothe pathological conditions characterized as inflammation. For example, iNOS-knockout mice are resistant to pleurisy and lung injury caused by carrageenan [28]. Selective inhibition of iNOS improves erosive joint disease [29], prevents experimental allergic encephalomyelitis [30], and attenuates immune dysfunction following trauma [8]. In addition, expression of iNOS has also been associated with various tumor types including brain, breast, lung, pancreas, liver, colon, and prostate cancers [9]. Selective NOS-2 inhibitors L-N6-(1-iminoethyl) lysine 5-tetrazole-amide (SC-51) and aminoguanidine (AG) actually show chemopreventive effect against the incidence of azoxymethane- (AOM-) induced colonic aberrant crypt foci [31], whereas NOS-2 blocker N-(3-(aminomethyl)benzyl) acetamidine (1400 W) is capable of suppressing tumor development in human colon adenocarcinoma DLD-1 xenograft [32]. These findings implicate the benefits of identifying novel agents targeting iNOS and its pertinent pathways. With this goal, we previously screened the ethanol extracts of 81 herbs for their ability to block LPS/IFN*γ*-induced inflammatory responses. Among them, we found that ethanol extract of* Artemisia anomala* S. Moore is effective to block LPS/IFN*γ*-induced NO production in RAW264.7 cells. In this study, we extracted ethanol extract of* Artemisia anomala* S. Moore further with four different solvents and found that ethyl acetate fraction (EAFA) had over 2-fold better potency than the original ethanol extract in the capability to inhibit NO production in LPS/IFN*γ*-stimulated RAW264.7 cells (Figure 1). Interestingly, EAFA also displayed significant protective effect to the viability of LPS/IFN*γ*-stimulated RAW264.7 cells without cytotoxicity in unstimulated cells (Figure 2). Our studies thus indicate the potential of using EAFA as anti-inflammatory effect.

Oxidative stress, which can arise from excessive ROS and/or RNS such as NO and its derivatives superoxide anions, may cause many diseases. The FRAP assay showed the antioxidative capacity of EAFA in inflammatory stimulation elicited cell damage as a cytoprotectant (Figure 4(a)). LPS/IFN*γ*-induced NO production is mediated by iNOS [22, 23]. We showed that EAFA blocked both iNOS mRNA and protein expression in activated macrophages (Figures 4(b) and 4(c)). HO-1 is the inducible isoform of the rate-limiting enzyme of heme degradation. Induction of HO-1 protects against the cytotoxicity of oxidative stress. HO-1 has been recognized to have anti-inflammatory properties [33]. EAFA induced the expression of HO-1 on mRNA and protein level (Figure 5). So, EAFA had dual properties in depressing the proinflammatory enzyme and inducing the anti-inflammatory enzyme.

Induction of iNOS is often accompanied with upregulation proinflammatory cytokines in macrophages [13, 14]. EAFA can also diminish the expression and secretion of IL-1*β* and IL-6 (Figure 5). The expression of a number of immunity and inflammatory related genes such as iNOS, IL-1*β*, and IL-6 was modulated by activated NF-*κ*B [34]. Under inflammatory conditions, inhibitory protein I*κ*Bs are promptly phosphorylated and degraded from p50 and p65 subunits binding site of NF-*κ*B; the activated NF-*κ*B subunits migrate to the nucleus. To investigate the possible preventive capability of EAFA on NF-*κ*B activation, we studied p50/p65 nuclear translocation by NF-*κ*B p50/p65 EZ-TFA transcription factor assay kit. LPS/IFN*γ* stimulate the activation of NF-*κ*B and induce p50/p65 movement to nucleus; EAFA repressed the amount of p50/p65 in the nucleus (Figures 6(a) and 6(b)). So EAFA displayed the interference in progress of NF-*κ*B active heterology dimmer heading to the nucleus.

MAPKs and NF-*κ*B signaling mechanisms have been previously linked to both iNOS and proinflammatory factor expression under inflammatory conditions. Moreover, several studies have shown that MAPKs play a critical role in the activation of NF-*κ*B [35]. Depending on the cell system, p38, ERK, and JNK have proven to have ROS-sensitive kinase activity [36]. According to the antioxidant activity of EAFA, we investigated whether MAPK pathway was involved in attenuating inflammatory mediators express and final NO/RNS reduction. In fact, our study showed that EAFA was able to abolish LPS/IFN*γ*-induced activation of Erk and JNK in RAW246.7 cells (Figure 6(c)). Together, we reason that the anti-inflammatory effect of EAFA is at least partly by attenuating NF-*κ*B and MAPKs activation.

In conclusion, our study indicates that EAFA can potently suppress inflammatory responses, and it hence warrants further identification of the effective component(s) in EAFA.

## Figures and Tables

**Table 1:** Primer sets for qRT-PCR.

Gene name	Forward primer	Reverse primer
iNOS	GGAGCGAGTTGTGGATTGTC	GTGAGGGCTTGGCTGAGTGAG
HO-1	CACAGATGGCGTCACTTCGTC	GTGAGGACCCACTGGAGGAG
IL-1*β*	GCTGTGGCAGCTACCTATGTCTTG	AGGTCGTCATCATCCCACGAG
IL-6	CCACTTCACAAGTCGGAGGCTTA	GTGCATCATCGCTGTTCATACAATC
*β*-actin	GCTACAGCTTCACCACCACAG	GGTCTTTACGGATGTCAACGTC

## Abbreviations

EAFA: Ethyl acetate extraction of* Artemisia anomala* S. Moore
LPS: Lipopolysaccharide
IFN-*γ*: Interferon-*γ*
NO: Nitric oxide
iNOS: Inducible nitric oxide synthase
HO-1: Inducible haemoxygenase
IL-6: Interleukin-6
IL-1*β*: Interleukin-1*β*
NF-*κ*B: Nuclear factor-kappa B
ERK: Extracellular signal-regulated kinase
JNK: c-Jun N-terminal kinase
MAPK: Mitogen-activated protein kinase.
